# Simulation of Land Use Change and Ecosystem Service Value Dynamics under Ecological Constraints in Anhui Province, China

**DOI:** 10.3390/ijerph17124228

**Published:** 2020-06-13

**Authors:** Sai Hu, Longqian Chen, Long Li, Ting Zhang, Lina Yuan, Liang Cheng, Jia Wang, Mingxin Wen

**Affiliations:** 1 School of Construction and Management, Jiangsu Vocational Institute of Architectural Technology, Xueyuan Road 26, Xuzhou 221116, China; saihu@cumt.edu.cn; 2 School of Environmental Science and Spatial Informatics, China University of Mining and Technology, Daxue Road 1, Xuzhou 221116, China; tingzhang@cumt.edu.cn (T.Z.); lnyuan@cumt.edu.cn (L.Y.); liang.cheng@cumt.edu.cn (L.C.); jia.wang@cumt.edu.cn (J.W.); mingxin.wen@cumt.edu.cn (M.W.); 3 Department of Geography, Earth System Science, Vrije Universiteit Brussel, Pleinlaan 2, 1050 Brussels, Belgium; long.li@cumt.edu.cn; 4 College of Yingdong Agricultural Science and Engineering, Shaoguan University, Daxue Road 26, Shaoguan 512005, China

**Keywords:** land use change, FLUS model, simulation, ecosystem service value

## Abstract

Land use change has a significant impact on the structure and function of ecosystems, and the transformation of ecosystems affects the mode and efficiency of land use, which reflects a mutual interaction relationship. The prediction and simulation of future land use change can enhance the foresight of land use planning, which is of great significance to regional sustainable development. In this study, future land use changes are characterized under an ecological optimization scenario based on the grey prediction (1,1) model (GM) and a future land use simulation (FLUS) model. In addition, the ecosystem service value (ESV) of Anhui Province from 1995 to 2030 were estimated based on the revised estimation model. The results indicate the following details: (1) the FLUS model was used to simulate the land use layout of Anhui Province in 2018, where the overall accuracy of the simulation results is high, indicating that the FLUS model is applicable for simulating future land use change; (2) the spatial layout of land use types in Anhui Province is stable and the cultivated land has the highest proportion. The most significant characteristic of future land use change is that the area of cultivated land continues to decrease while the area of built-up land continues to expand; and (3) the ESV of Anhui Province is predicted to increase in the future. The regulating service is the largest ESV contributor, and water area is the land use type with the highest proportion of ESV. These findings provide reference for the formulation of sustainable development policies of the regional ecological environment.

## 1. Introduction

Land resources are an important foundation for human survival and development [1,2]. As human activity intensifies, the structure and functions of land use have undergone significant changes [3,4,5]. However, the development of social economy and huge population pressures have promoted the increase in the intensity of land resource use [6], which has led to a series of ecological and environmental problems, and the damage to ecosystems has threatened global ecological security and sustainable development [7,8,9]. With the enhancement of human’s understanding of ecosystems and awareness of ecological protection, the estimation of ecosystem service value (ESV) has become an important foundation for the implementation of ecosystem management and a hotspot for regional ecological environment evaluation research [10,11,12].

The land use change simulation model is an effective tool for regional future land use layout simulation and provides technical support for the formulation of land use planning [13,14]. With the development and application of computer science and geographic information system (GIS) technology, research on land use spatial layout simulation models has rapidly increased [15,16,17,18]. Researchers have used a variety of simulation models to simulate future land use in terms of the quantitative characteristics and spatial layout, including a multi-agent system (MAS) model [19], cellular automata (CA) model [20], the conversion of land use and its effects at a small regional extent (CLUE-S) model [21], and a future land use simulation (FLUS) model [22], etc. The FLUS model, first proposed by Liu et al. [14], can effectively deal with the complexity and uncertainty of the conversion of various land use types under the mutual influence of the human-land relationship. The model is mainly used in land use simulation, urban expansion simulation, and the delineation of urban growth boundaries [13,22,23,24,25]. The model adds an adaptive inertial competition mechanism based on the traditional CA model. In the simulation process, the model uses inertia coefficients to represent the characteristics of different land types, and select the final conversion type using a roulette selection method, which improves the prediction accuracy of land use patterns [26]. There are many possibilities for future land use changes due to the impact of policies, population, and economic development. The FLUS model is an integrated model that can combine human activities and environmental effects to simulate land use changes in different scenarios, such as the natural growth scenario, urban expansion scenario, and ecological security scenario [27].

Ecosystem services refer to the natural environmental conditions and functions of ecosystems and ecological processes that maintain human survival [28]. Land use change affects the structure and function of ecosystems, and ESV estimation is an important method to evaluate the ecological effects of land use change [29]. Many studies on the estimation of multiple ESVs at different scales have been implemented using different methods [30,31,32,33]. According to the existing literature, the commonly used estimation methods of ESVs can be summarized as the emergy analysis method, ecological space evaluation method, material quality evaluation method, and the value quantity evaluation method [34]. There are relatively fewer restrictions for the value coefficient method based on the land use type in terms of the estimation cost, estimation time, and difficulty of data acquisition, and it is relatively simple to operate, which is conducive to promoting the extensive use of this method [35]. In 1997, the ESV estimation model proposed by Costanza et al. [36] was generally recognized and used, which played a positive role in promoting the study of ESV estimation. On this basis, Xie et al. [37] revised the estimation model according to the ecosystem characteristics in China, and their model has been widely used in China. However, this revised estimation model is aimed at nationwide ecosystems and is suitable for large-scale ESV studies [28]. The ecosystem of China is complex, and the level of socio-economic development varies greatly in different provinces [38]. Applying this model directly to small to medium scale ESV estimation research may reduce the estimation accuracy. Therefore, it is necessary to revise this model based on the actual situation of the study area to improve the applicability and estimation accuracy.

It is obvious that the future land use change simulations to assess the potential impact on the ecological environment have become a research hotspot [39]. Although previous studies have proved the effectiveness of the FLUS model in future land use simulations [40,41,42,43], there is still a lack of land use change simulation researches based on ecological constraints to improve the ESV. Therefore, we expect to construct an ecological optimization scenario to simulate future land use change and increase the ESV, which aims at providing the government with appropriate and effective suggestions to improve the sustainability of land use and ecological protection policies.

The rapid urbanization process in Anhui Province has led to growing population and land use demand over the past few decades. As a result, the regional land use structure has changed significantly and caused various environmental problems such as land degradation, water quality deterioration, and biodiversity reduction, which have affected the sustainable development of Anhui Province [44]. Therefore, it is necessary to optimize the land use structure and estimate the ESV to promote ecological environmental protection and sustainable development. In order to achieve this goal, three steps were taken here: First, the simulation scenario for optimization of land use structure was constructed under ecological constraints; then, the land use structure and layout of Anhui Province in 2025 and 2030 were simulated using the FLUS model; finally, the ESVs of Anhui Province since 1995 were estimated using the revised estimation model.

## 2. Materials and Methods

### 2.1. Study Area

**Figure 1:**
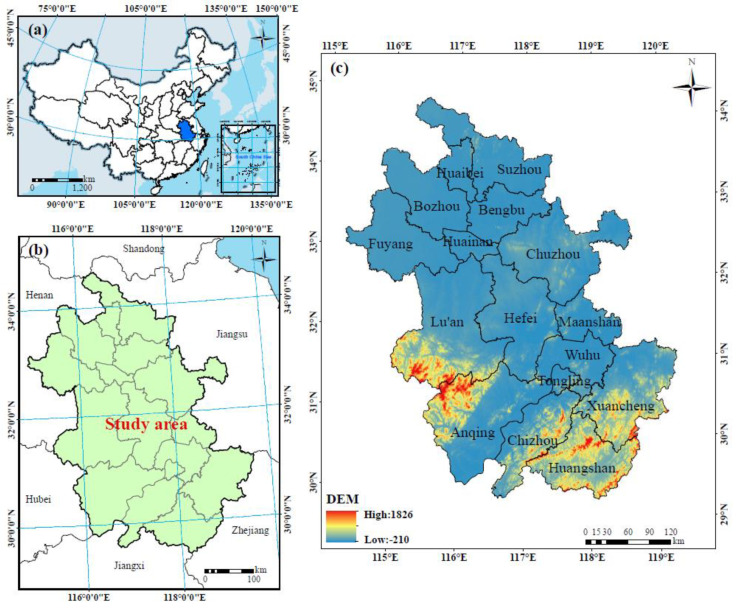
The study area: (**a**) the location of Anhui Province in China; (**b**) the administrative division of Anhui Province; (**c**) the digital elevation model (DEM) of Anhui Province.

Anhui Province is located in the lower reaches of the Yangtze River (114°54′–119°37′ E, 29°41′–34°38′ N), across the Huaihe River in eastern China, and it is an important part of the Yangtze River Delta urban agglomeration (Figure 1). Anhui Province has a low average elevation, and the terrain is complex and diverse. The region has a warm and humid climate, with an average annual temperature between 14–17 °C, and an average annual precipitation between 800–1800 mm. The population of Anhui Province was 63.24 million in 2018, and the urbanization rate reached 54.69%. With a fast economic development rate, the gross domestic product (GDP) of the province reached 41,979.51 × 10^7^ USD (United States dollars) in 2018, an increase of 8.02% over 2017. Driven by the rapid social and economic development, the land use structure in Anhui Province has undergone great changes, especially a significant increase of the area of built-up land.

### 2.2. Data

All the satellite data were downloaded from the United States Geological Survey (USGS) website (http://earthexplorer.usgs.gov), including images from Landsat 5 TM (Thematic Mapper), Landsat 7 ETM+ (Enhanced Thematic Mapper Plus), and Landsat 8 OLI (Operational Land Imager), with a 30 m spatial resolution (Table 1). We obtained the land use data in 1995, 2000, 2005, 2010, 2015, and 2018 by interpreting remote sensing images using Maximum Likelihood Classification (MLC) method in the ENVI 5.1 Classic remote sensing image processing software package, which were classified into 8 types (paddy field, unirrigated field, forest land, grass land, water area, wet land, built-up land, and unused land). High-resolution satellite images from Google Earth Pro were used to assess the accuracy of the land-use classification by constructing a confusion matrix. We calculated the classification accuracies by programming in MATLAB. Because the high-resolution images in 1995 and 2000 were not available in Google Earth Pro, we only s verified the classification results for 2005, 2010, 2015, and 2018. The overall accuracies for 2005, 2010, 2015, and 2018 were 87.30%, 86.50%, 86.90%, and 85.10%, respectively; the kappa coefficients were 0.8529, 0.8355, 0.8372, and 0.8120, respectively. The classification results were rather acceptable as the kappa coefficients are greater than 0.8.

The digital elevation model (DEM) data, with a spatial resolution of 30 m, were downloaded from the Geospatial Data Cloud (http://www.gscloud.cn). The kilometer grid data of the population density and absolute GDP in 2015 and 2018 were collected from the National Earth System Science Data Center (http://www.geodata.cn). The meteorological data include the annual average rainfall data and the annual average temperature data of Anhui Province in 2015 and 2018 as provided by the National Meteorological Information Center (https://data.cma.cn). The soil type data were obtained from the Resource and Environment Data Cloud Platform (http://www.resdc.cn), and the soil attribute data includes the content of sand, silt, clay, soil organic matter, and gravel, which were obtained from the Harmonized World Soil Database version 1.2 (http://westdc.westgis.ac.cn). The socio-economic statistics were obtained from the Statistical Yearbooks of China and Anhui Province, including the population, GDP, urbanization rate, Engel’s coefficient, grain production, and the average price of grain.

### 2.3. Methods

**Figure 2:**
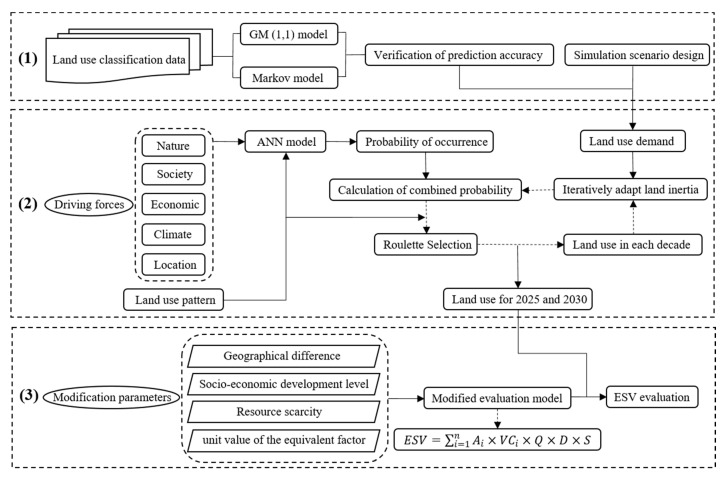
The framework of this study: (**1**) prediction of land use structure; (**2**) Land use layout simulation using FLUS model; (**3**) ESV estimation using the revised model.

We developed the framework (Figure 2) for this paper in accordance with the research objectives here. First, we predicted the land use structure based on the land use classification data using the grey prediction (1,1) model (GM) and Markov model, selecting the model with a relatively high-precision to predict the land use structure in the future. In addition, we designed a simulation scenario as the constraint on the simulation of the land use spatial layout to optimize the structure of ecosystem. Then, the FLUS model was used to simulate the spatial layout in 2025 and 2030 under the ecological optimization scenario using the GeoSOS-FLUS software that developed according to the principle of FLUS model: the probabilities of occurrence for each land use type will be obtained using artificial neural network (ANN) model combined with driving forces of land use change and land use pattern; the cellular automata (CA) model and the calculated data of land use demand and probabilities of occurrence will be used to simulate future land use spatial layout data. Finally, we modified the ESV evaluation model that considering the ecological and economic spatial heterogeneity (including geographical difference, socio-economic development level, resource scarcity, and regional ecosystem difference) and evaluated the ESV dynamics based on the simulation results of land use change.

#### 2.3.1. Land Use Structure Prediction

It is necessary to determine the demand for each land use type before simulating the land use spatial layout [14]. According to previous literature, commonly used land use demand prediction models include GM (1,1) model [45], Markov model [46], and System Dynamics (SD) model [47]. In order to ensure the prediction accuracy of land use demand, different prediction models will be used to predict the land use demand, respectively. By comparing the prediction accuracy, the model with relatively high precision will be selected to predict the future land use demand. From the perspective of model operation, we can directly obtain future land use demand data using the GM (1,1) model and Markov model, which only requires previous land use data. However, a large number of variables and long time series data are required to predict future land use demand using the SD model [48], which is difficult in data collection and model operation. Therefore, the GM (1,1) model and Markov model were selected for land use demand predicting and accuracy comparison in this paper.

The grey system theory proposed by Chinese scholar Deng [49] is widely used in economics, geography, agriculture, and other fields for prediction, decision-making, and evaluation [50,51,52], which has no strict requirements on the selected sample data and is easily operated [45,53]. The methods for verifying the accuracy of prediction results include residual test, correlation test, and posterior difference test. In this study, the commonly used posterior difference test was selected to test the prediction accuracy of the GM (1,1) model. The indicators used in the test are the posterior difference ratio and the small error possibility, and the prediction accuracy is divided into four levels, as shown in Table 2. The prediction process of the GM (1,1) model was implemented in the MATLAB software package.

The Markov model is constructed on the basis of stochastic process theory [46], which can predict the future status of an event, only requiring the data concerning the current time and a previous time [54]. The Markov model has advantages for predicting future land use change, because the continuous historical data are not required. The mathematical expression of the Markov model is given as follows:

(1) $$S_{t+1}=P_{ij}*S_{t},$$

where $S_{t}$ represents the status of land use types at the current time, $S_{t+1}$ represents the status of future land use types, $P_{ij}$ represents the transition probability matrix for land use types:

(2) $$P_{ij}=\left[\begin{array}{cc} P_{11} & P_{12}\\ \begin{array}{c} P_{21}\\ \cdots \\ P_{n1} \end{array} & \begin{array}{c} P_{22}\\ \cdots \\ P_{n2} \end{array} \end{array}\begin{array}{cc} \cdots & P_{1n}\\ \begin{array}{c} \cdots \\ \cdots \\ \cdots \end{array} & \begin{array}{c} P_{2n}\\ \cdots \\ P_{nn} \end{array} \end{array}\right],$$

where 0 ≤ $P_{ij}$ ≤ 1, and i, j = (1, 2, 3, …, n).

#### 2.3.2. Simulation Scenario Setting

The direction of land use change in the future is uncertain [55]. Many studies on the simulation of land use change under different scenarios exist, including natural growth scenario, urban expansion scenario, and ecological protection scenario [22]. There are no restrictions on the conversion of land use types in the natural growth scenario, and this scenario is in full accordance with the natural law of land use type evolution. In order to meet the needs of population growth and industrial development, priority is given to ensuring the expansion of built-up land in the urban expansion scenario. The ecological protection scenario aims to take strict ecological protection measures against ecological problems such as environmental damage and land degradation and to improve the ecological environment. In this paper, we construct an ecological optimization scenario and determine the conversion rules of land use types based on a comprehensive consideration of the possibility of land use development and ecological protection.

**Figure 3:**
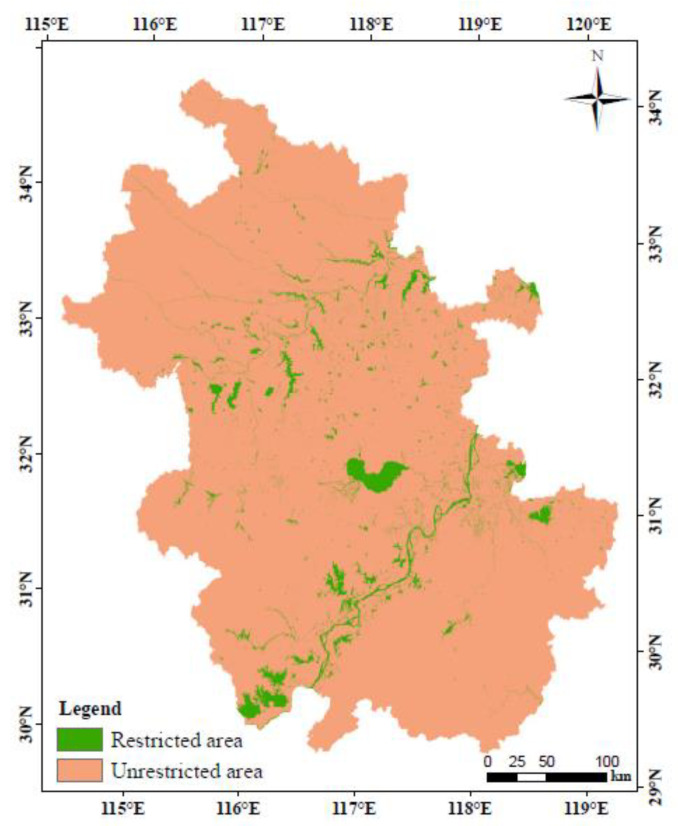
Restricted area for land use conversion. Green part is the water area that is restricted to be transformed, while land type conversion in the other part is allowed.

First, land use types with the highest ESV are not allowed to be converted into other land use types. In our previous research, water area was proven to be the land use type with the greatest ESV. Therefore, we set the water area as the restricted area here (Figure 3).

Land use types with a relatively high ESV (including wet land, forest land, and grass land) are not allowed to be converted into the land use types with a relatively low ESV (including built-up land, paddy field, and unirrigated field). This means that reverse conversions are not allowed. Finally, the appropriate expansion of built-up land is allowed based on the consideration of socio-economic development. In the conversion rules, there are no restrictions on the conversion of paddy field and unirrigated field, but it is necessary to ensure that the total area of paddy field and unirrigated field is larger than their protection area. Under the above principles, the rules of land use types conversion under the ecological optimization scenario of the study area were obtained and shown in Table 3.

#### 2.3.3. Land Use Spatial Layout Simulation

The FLUS model is used to simulate future land use change based on CA and ANN [14]. The GeoSOS-FLUS model software, developed based on the FLUS model, is an effective tool for geospatial simulation, participation in spatial optimization, and assistance in decision-making [56]. The software includes two modules, an ANN-based suitability probability estimation module and a self-adaptive inertia and competition mechanism CA module.

The ANN-based suitability probability estimation module combines land use data with the driving forces of land use change and uses the ANN to obtain the suitability probability of each type of land use in the study area [41]. The driving forces of land use change include human activities and natural effects [57]. The most important driving force of land use change in the short to medium term is socio-economic factors, represented here by the GDP and population density [58]. Natural environment factors affect the spatial distribution characteristics of land use, especially at large scales [59]. In addition, traffic location factors play an important guiding role in the future land use change. Finally, we selected 16 driving factors from socio-economic, natural environment, and traffic location factors (Table 4).

**Figure 4:**
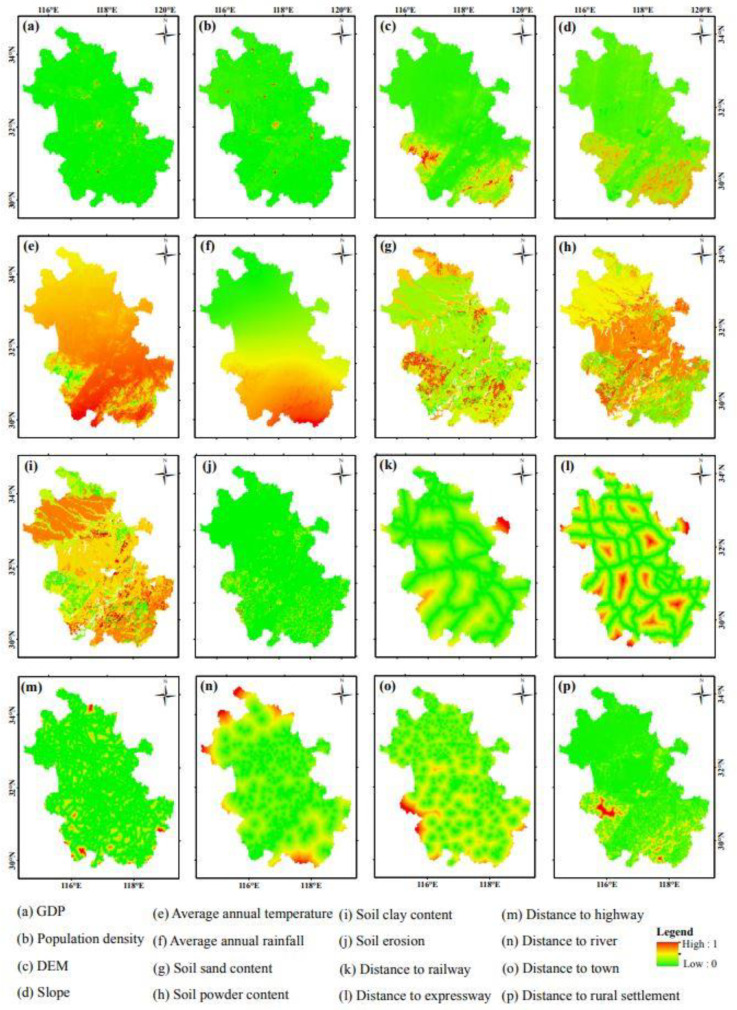
Raster map of land use change driving factors for the FLUS model.

All the data of the driving factors were normalized and exported as raster type data (Figure 4). The data of the GDP, population density, DEM, and soil attribute data were obtained by resampling the original data. Slope data were obtained by performing slope analysis on the DEM data using the ArcGIS software package. The annual average temperature and annual rainfall data were obtained using spatial interpolation analysis combined with meteorological station data. Soil erosion data were calculated using the improved soil loss estimation model [60]. Traffic location data were obtained using the distance analysis tool in ArcGIS.

The binary logistic regression model is a probabilistic nonlinear regression method for predicting the relationship between binary classification results and multiple influencing factors [61], which was used to test the relationship between the driving factors and land use change to determine the explanatory power of various driving factors in this paper. The receiver operating characteristic curve (ROC) is a commonly used method for checking the calculation results of the logistic regression model [62]. According to the principle of ROC, the curve is drawn using the movement of cutoff point combined with the calculation results of the logistic regression model. The larger the area under the curve, the higher the explanatory value of the independent variable to the dependent variable.

The range of the ROC values varies from 0 to 1. The closer the ROC value is to 1, the more accurate the prediction result of the logistic regression model is. If the ROC value is less than 0.5, it means that the predictive ability of the model is low. The ROC values were calculated by the analysis module of SPSS 22.0 software based on the regression coefficients of the driving factors and land use types (Table 5). The results show that only the ROC values of grass land and built-up land are less than 0.9, and the other ROC values are greater than 0.9. Therefore, we believe that all the driving factors are suitable for estimating the suitability probability.

#### 2.3.4. Estimation of Ecosystem Service Value

The estimation method of ESV combined with the equivalent factor of the ESV per unit area was developed and improved by Xie et al. [37] based on the research of Costanza et al. [36]. This method is less restricted by estimation cost, estimation time, and data acquisition, which is suitable for regional or large-scale ESV estimation. In addition, the method is developed according to the characteristics of the ecosystem in China. Therefore, it has been widely used and in China [63] and was selected as the basis for this study to estimate ESV. The estimation method is given by the following equation:

(3) $$ESV=\overset{n}{\underset{i=1}{\sum }}A_{i}\times VC_{i},$$

where $A_{i}$ is the area of the *i*th land use type and $VC_{i}$ is the ESV per unit area of the *i*th land use type.

In our previous research, we adjusted the equivalent value of ecosystem service value per unit area of terrestrial ecosystem according to the ecological characteristics of Anhui Province [28], and calculated the ESV coefficients of each land use type using the following equation:

(4) $$E_{a}=\frac{1}{7}\times p\times \frac{1}{n}\overset{m}{\underset{i=1}{\sum }}Q_{i},$$

where $E_{a}$ is the unit value of the equivalent factor (USD/ha), p is the average grain price (USD/kg), and Q is the annual average grain yield (kg/ha).

In this study, we selected rice, wheat, corn, beans, and potatoes to calculate the annual average grain yield in Anhui Province, which is 4798.71 kg/ha. In order to eliminate the influencing factors such as food price fluctuations and currency inflation in different research periods on the estimation results, we used the average food price in 2018 (USD/kg) as the unified price and calculated the unit value of the equivalent factor, which is 262.34 USD/ha. Finally, we calculated the ESV coefficients of each land use type in Anhui Province (Table 6).

In order to improve the applicability of the estimation model in the study area, we further revised the estimation model based on the spatial heterogeneity.

Since the equivalent factor value we used is defined by the economic value of the annual grain yield in a 1 ha farmland area with an average national yield, we need to convert the equivalent factor value at the national scale into the scale of the study area. We used the ratio of the average grain yield in the study area to the national average grain yield as the grain yield correction factor using the equation as follows:

(5) $$Q=\frac{G_{A}}{G_{N}},$$

where Q is the grain yield correction factor, $G_{A}$ is the average grain yield of the study area (kg/ha), and $G_{N}$ is the national average grain yield (kg/ha).

Human beings will pay more attention to the protection of ecosystems and ecological environments with the development of economy, which means that investment in ecological environmental protection and management will continue to increase [64]. In order to make the evaluation of ESV consistent with the level of socio-economic development, we added the socio-economic development correction factor. It is given by the following equation:

(6) $$D=P_{w}\times P_{v},$$

where D is the socio-economic development correction factor, $P_{w}$ is the willingness to pay, and $P_{v}$ is the ability to pay.

The Engel’s coefficient indicates the proportion of total food expenditure to total personal consumption expenditure. With a decrease of the Engel’s coefficient, people are more willing to spend money on non-food consumption. Therefore, we use the Engel’s coefficient to measure the willingness to pay and obtain the stage coefficient of socio-economic development, which was calculated using the Peal growth curve model as follows:

(7) $$P_{w}=\frac{L_{A}}{L_{N}},$$

(8) $$L=\frac{1}{1+e^{-\left(\frac{1}{EL}-3\right)}},$$

where $L_{A}$ is the stage coefficient of the socio-economic development of the study area, $L_{N}$ is the national stage coefficient of socio-economic development, and EL is the Engel’s coefficient (%).

The GDP is consistent with the economic development level and can represent the ability to pay. A large number of the agricultural population flow into cities in the process of urbanization, which has led to changes in the regional production structures, lifestyles, landscape patterns, social welfare benefits, and price levels [65]. This means that people’s ability to pay has also changed. Therefore, we chose the GDP per capita and urbanization rate to measure the ability to pay in the study area:

(9) $$P_{v}=\frac{pGDP_{A}}{pGDP_{N}}\times \frac{U_{A}}{U_{N}},$$

where $pGDP_{A}$ is the GDP per capita of the study area, $pGDP_{N}$ is the national GDP per capita, $U_{A}$ is the urbanization rate of the study area, and $U_{N}$ is the national urbanization rate.

Resource scarcity reflects the relationship between the supply and demand of regional ecological resources [66]. Under the condition that the supply of ecological resources is constant or reduced, the greater the demand for an ecological resource, the higher the of ecological resource scarcity, and the willingness to pay for ecological resource will also increase. There is a significant linear correlation between the demand for ecological resources and the total population, so we use population density to measure resource scarcity here:

(10) $$S=\frac{\ln P_{A}}{\ln P_{N}},$$

where S is the resource scarcity correction factor, $P_{A}$ is the population density of the study area, and $P_{N}$ is the national population density.

Finally, we obtained the revised ESV estimation model as follows:

(11) $$ESV=\overset{n}{\underset{i=1}{\sum }}A_{i}\times VC_{i}\times Q\times D\times S,$$

## 3. Results

### 3.1. Prediction Results of Land Use Structure

According to the principle of the GM (1,1) prediction model, we obtained the land use structure in 2015 and 2018 to first verify the prediction accuracy (Table 7). We did not predict the area of unused land due to its small area, and we believe that all unused land will convert into built-up land with the process of urbanization. The prediction results show that the maximum difference is for wet land, which are 4.56% and 2.52% in 2015 and 2018, respectively. The prediction results of other land use types are all below 1.3%, and the minimum value is −0.05% for built-up land in 2018.

The land use prediction accuracy of the GM (1,1) model was obtained using the accuracy verification method and the prediction results (Table 8). According to the calculation results of the posterior difference ratio and the small error possibility, we determined the prediction accuracy levels for each land use types. The prediction accuracies of all land use types are qualified (greater than level 4) and most of the accuracy levels are level 1 or level 2, altough the prediction accuracy of wet land is at level 3, but this is acceptable.

We also predicted the land use structure using the Markov model and calculated the prediction accuracy (Table 9). The differences greater than 5% in 2015 include water area, wet land, and built-up land, of which the maximum value is −12.82%. The prediction accuracy in 2018 has improved, and the maximum difference is −4.77% for built-up land.

By comparing the prediction results of the two models (Table 10), we find that almost all the prediction accuracies of the Markov model for each land use type are lower than that of the GM (1,1) model, of which the most significant difference is 11.63 of built-up land in 2015. It can be considered that the GM (1,1) model has a higher prediction accuracy in the study area by comparing the prediction results of the two models. Therefore, we used the GM (1,1) model to predict the land use structure in the study area in 2025 and 2030.

Table 11 presents the prediction results of land use structure in Anhui Province for 2025 and 2030. By comparing the land use structures for 2025 and 2030, we find that the areas of built-up land and water area show an increasing trend, while the other land use types show a decreasing trend.

### 3.2. Simulation Results of Land Use Spatial Layout

#### 3.2.1. Accuracy Verification of FLUS model

In order to verify the simulation accuracy of the FLUS model, we simulated the land use spatial layout for 2018 based on the land use data of 2015, and compared the results with the actual land use data for 2018 (Table 12). We randomly sampled the simulated raster data at a ratio of 1%, and calculated the overall accuracy and kappa coefficient, which were 91.75% and 0.8935, respectively. This result indicates that the model has high simulation accuracy and can be used to simulate future land use change in the study area.

#### 3.2.2. Land Use Spatial Layout Simulation

**Figure 5:**
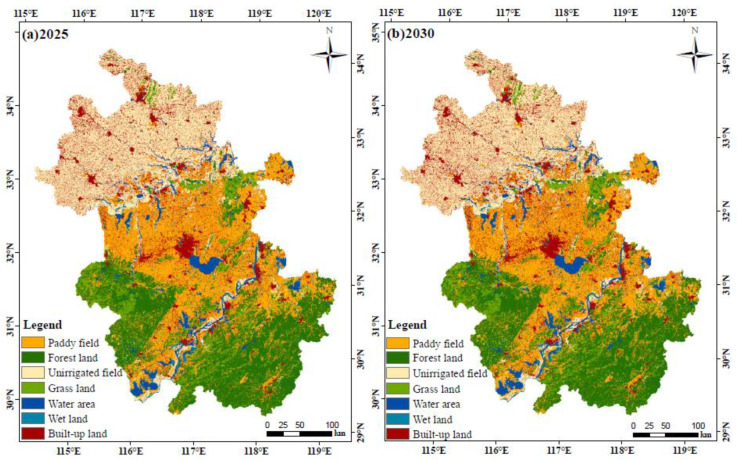
Simulation results of land use spatial layout in (**a**) 2025 and (**b**) 2030 using the FLUS model.

Figure 5 shows the simulation results of the land use spatial layout under the ecological constraint of Anhui Province. We can see that the spatial layout of land use types for 2025 and 2030 in Anhui Province is stable and that cultivated land is still the main land use type. In order to analyze the trend of land use change, we calculated the area and proportion of each land use type for 2025 and 2030 (Table 13). Compared with the land use structure in 2018, the area of paddy field, unirrigated field, grass land, and wet land will decrease in the future. In addition, the area of forest land, water area, and built-up land shows an increasing trend, of which the growth rate of built-up land is 2.56% and 9.45% in 2025 and 2030, respectively.

### 3.3. Change Characteristics of ESV

**Figure 6:**
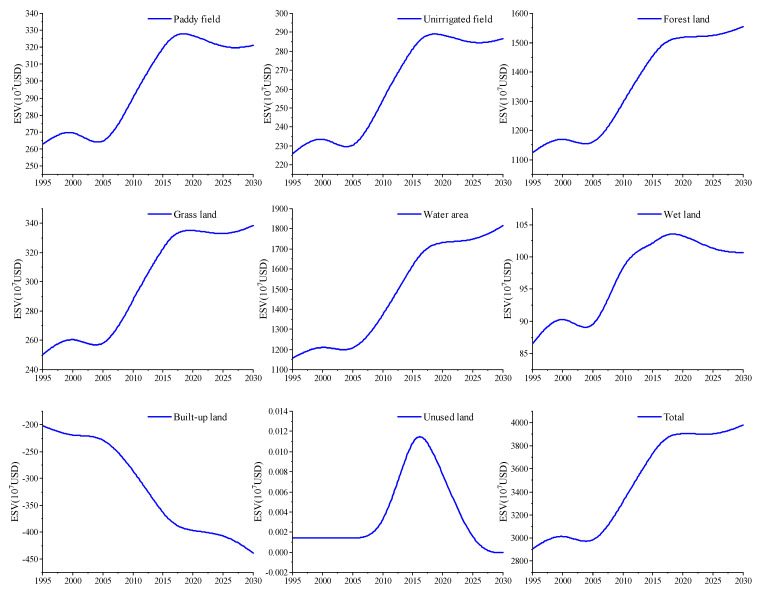
ESV change trends of various land use types from 1995 to 2030.

We estimated the ESVs of all land use types in Anhui Province from 1955 to 2030 and these estimations are presented in Table 14. The estimation results show that the total ESV is predicted to increase from 2906.65 × 10^7^ USD in 1995 to 3979.11 × 10^7^ USD in 2030, and the growth rate is 36.9%. Water area is the land use type with the highest proportion of ESV, with a proportion exceeding 40%. The ESV proportion of forest land is close to water area, accounting for about 40% of the total value, and the proportions of the other land use types are below 10%. Figure 6 shows the change trend of ESVs for different land use types. We can see that the ESV changes of paddy field, unirrigated field, forest land, grass land, water area and wet land show a fluctuating growth trend that is consistent with the total ESV. Under the ecological optimization scenario, the ESVs of paddy field, unirrigated field and wet land will decrease in 2025 and 2030. Obviously, the contribution of built-up land to the ESV is negative and is predicted to decrease from −201.47 × 10^7^ USD in 1995 to −438.53 × 10^7^ USD in 2030, showing a decreasing trend. Because the ESV of unused land is extremely low, and we believe that it will be converted into built-up land in the future, we have not analyzed it in this paper.

**Figure 7:**
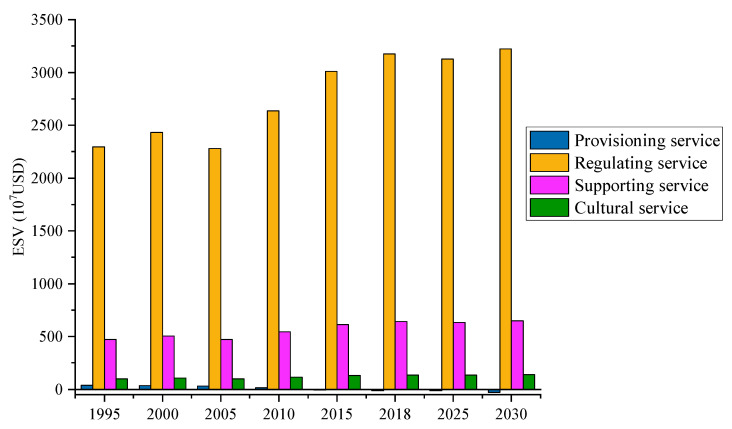
Changes in ESVs of different ecosystem service types from 1995 to 2030.

In addition, we also calculated the ESVs of different ecosystem service types (Figure 7). The regulation service is the largest ESV contributor in the study area, accounting for about 80% of the total ESV, followed by supporting service, which accounts for more than 16% of the total ESV. The ESVs of the cultural service and provisioning service account for small proportions of the total ESV, and all of them are below 4%. From the change trend perspective, the ESV of the regulation service shows a fluctuating growth trend, which is predicted to increase from 2296.56 × 10^7^ USD in 1995 to 3224.16 × 10^7^ USD in 2030, with a growth rate of 40.39%. Although the ESVs of the supporting service and cultural service show an increasing trend from 1995 to 2030, the increments are only 173.91 × 10^7^ USD and 37.80 × 10^7^ USD. However, the ESV of the provisioning service continues to decrease and will decrease to −29.83 × 10^7^ USD by 2030, which has a negative contribution to the total ESV.

## 4. Discussion

### 4.1. Characteristics of Land Use Change

The simulation results show the characteristics of land use layout from 2018 to 2030. From the perspective of land use change, the most significant characteristic is that the area of cultivated land (including paddy field and unirrigated field) continues to decrease, while the area of built-up land continues to increase, which is a manifestation of increased human interference and urbanization [65]. The periphery of cities and counties are hot spots for these land use conversion types. In the context of rapid urbanization, a large number of migrant workers have flowed into the city and led to increased demand for urban built-up land, such as residential land, transportation land, and recreational facility land [67]. With the development of urbanization and the adjustment of industrial structure, the demand for industrial production land will increase rapidly, which provides more employment opportunities for people. A higher quality of life and more employment opportunities will further promote the population agglomeration and the expansion of built-up land in the city [28], leading to a continuous circular promotion process.

### 4.2. Applicability of the Simulation Model

In this paper, we simulated the land use change of Anhui Province in 2018 using the FLUS model. By comparing the spatial layout of each land use type, we found that the simulation results are reasonable, which shows that it is feasible to use the FLUS model to simulate future land use change. The FLUS model uses an adaptive inertial competition mechanism based on roulette selection, which can effectively deal with the uncertainty and complexity of the mutual conversion of land use types under the interaction of the natural environment and human activities [14]. In addition, we can set different conversion rules according to the simulation scenario when we use this model for simulation, which plays an active role in the optimization of land use structures [24]. We constructed an ecological optimization simulation scenario to restrict the conversion of land use types with a higher ESV coefficient to those with a lower ESV coefficient using the conversion matrix function of the FLUS model, in order to increase the ESV and improve the ecological environment.

### 4.3. Measurement of ESV in Anhui Province

The ESV coefficients of water area is much higher than the coefficients of other land use types. This is why the ESV of water area accounts for the highest proportion of the total value in Anhui Province, although the corresponding area is relatively small. In addition, the relatively high ESV coefficient and relatively large area make the ESV of forest land second to that of the water area. Mutual conversion between different land use types is the direct cause of ESV change. The most significant characteristic of land use structure change in Anhui Province is the continuous expansion of built-up land. Because the ESV coefficient of built-up land is smaller than that of all the other land use types, the conversion of built-up land to the other land use types will lead to the growth of ESV, while the conversion of all land use types to built-up land will lead to negative growth of ESV. Therefore, we believe that with the progress of urbanization, the rapid growth of built-up land has an increasingly negative impact on ESV [68]. However, the ecological optimization scenario we constructed will play a positive role in protecting the land use types with a high ESV coefficient and help reduce the loss of ESV in Anhui Province.

### 4.4. Innovation and Limitation

This study constructed an ecological optimization scenario to restrict the conversion of land use types with higher ESVs to that with lower ESVs, with the goal of improving future ESV. Based on this scenario, the future land use structure obtained by FLUS model effectively protects the land use types with high ESV coefficients, which obviously helps to improve the ESV. The ESV estimation model of Xie et al. [37] aims at a nationwide ecosystem. China has a vast territory, and the differences in environmental resources and socio-economic development of each region are obvious [28]. Therefore, we revised this model and improved it in accordance with the actual environment of the study area. We have added the grain yield correction factor, socio-economic development correction factor, and the resource scarcity correction factor to revise the estimation model, which makes up for the lack of consideration of spatial heterogeneity in the original model and helps to estimate the ESV more accurately.

Although the FLUS model can effectively simulate the land use changes in the short to medium term, there are still some uncertainties in the long-term prediction. For a long research period, the impact of the climate, human activities, and other driving factors on land use change is difficult to determine [63,69], which has also become a challenge for land use change simulation. Therefore, we can use different models for land use change simulation in future research and compare the results to improve the simulation accuracy. We know that there are great uncertainties and policy orientations for future regional development, and the development goals of each region are different [70]. We can construct different land use change simulation scenarios to adapt to the multiple possibilities of regional development in subsequent research. In addition, we have estimated the ESVs based on the revised estimation model and obtained some meaningful research results. However, it is worth further evaluating the applicability of the revised model. Nonetheless, this does not affect the final results of this study and can provide support in the formulation of land use and ecological protection policies.

## 5. Conclusions

In this study, we have simulated the dynamic changes of land use structure in Anhui Province under the ecological optimization scenario for 2025 and 2030, and the models used include the GM (1,1) model and FLUS model. In addition, we have estimated and analyzed the ESV change characteristics of Anhui Province since 1995 based on the revised ESV estimation model. The key findings and main conclusions are summarized as follows:

(1) The prediction accuracy of the land use demand of Anhui Province by the GM (1,1) model is higher than that of the Markov model, and the simulation errors of all land use types are within 5%. We simulated the land use spatial layout in 2018 based on the land use data of Anhui Province in 2015. The simulation results show that the overall accuracy is 91.75% and that the kappa coefficient is 0.8935, indicating that the FLUS model is applicable for land use layout simulation in Anhui Province.
(2) Under the ecological optimization scenario, the land use structure of Anhui Province is relatively stable. By 2030, the proportion of cultivated land is predicted to decrease to 52.78%, but it is still the predominant land use type in Anhui Province. The area of built-up land shows a significant expansion trend, and the proportion is predicted to reach 12.51% in 2030. In addition, the area of grass land and wet land will decrease in the future, while the area of forest land and water area will increase.
(3) The ESV of Anhui Province shows a fluctuating growth trend, which is predicted to increase from 2906.65 × 10^7^ USD in 1995 to 3979.11 × 10^7^ USD in 2030, with a growth rate of 36.9%. The water area has the highest ESV, with a proportion exceeding 40%, followed by forest land, with an ESV proportion approaching 40%. According to the contributions of the four ecosystem service types to ESVs, we sorted them in the order of regulating service > supporting service > cultural service > provisioning service, of which the ESV proportion of the regulating service is about 80%.

In summary, the FLUS model is applicable for simulating the future dynamic changes of land use in the short to medium term, which has a positive significance for the government to develop and execute land use planning. The results of this study may also help decision-makers to evaluate the development mode of land use to optimize the land use structure and promote the sustainable development of the ecological environment.

## Figures and Tables

**Table 1:** Landsat image data used in this study.

Year	Sensor	Acquisition Date (Path/Row)
1995	TM	1995-10-13 (120/37), 1995-10-13 (120/38), 1995-10-13 (120/39), 1995-10-13 (120/40),
		1995-10-05 (121/36), 1995-10-05 (121/37),1995-10-05 (121/38), 1995-10-05 (121/39),
		1995-10-11 (122/36), 1995-10-11 (122/37), 1995-10-11 (122/38), 1995-10-11 (122/39),
		1996-10-20 (123/36), 1996-10-20 (123/37)
2000	TM	2000-10-10 (120/37), 2000-10-10 (120/38), 2000-10-10 (120/39), 2000-10-10 (120/40),
		2000-11-02 (121/36), 2000-11-02 (121/37), 2000-11-02 (121/38), 2000-11-02 (121/39),
		2000-09-22 (122/36), 2000-09-22 (122/37), 2000-10-08 (122/38), 2000-10-08 (122/39),
		2000-10-15 (123/36), 2000-10-15 (123/37)
2005	TM	2005-10-24 (120/37), 2005-10-24 (120/38), 2005-10-24 (120/39), 2005-10-24 (120/40),
		2005-10-31 (121/36), 2005-10-31 (121/37), 2005-10-31 (121/38), 2005-10-31 (121/39),
		2005-11-07 (122/36), 2005-11-07 (122/37), 2005-11-07 (122/38), 2005-11-07 (122/39),
		2005-10-29 (123/36), 2005-10-29 (123/37)
2010	ETM+	2010-10-30 (120/37), 2010-10-30 (120/38), 2010-10-30 (120/39), 2010-10-30 (120/40),
		2010-10-05 (121/36), 2010-10-05 (121/37), 2010-10-05 (121/38), 2010-10-05 (121/39),
		2010-10-28 (122/36), 2010-10-28 (122/37), 2010-10-28 (122/38), 2010-10-28 (122/39),
		2010-11-04 (123/36), 2010-11-04 (123/37)
2015	OLI	2015-10-20 (120/37), 2015-10-20 (120/38), 2015-10-20 (120/39), 2015-10-20 (120/40),
		2015-10-11 (121/36), 2015-10-11 (121/37), 2015-10-11 (121/38), 2015-10-11 (121/39),
		2015-10-02 (122/36), 2015-10-02 (122/37), 2015-10-02 (122/38), 2015-10-02 (122/39),
		2015-10-09 (123/36), 2015-10-09 (123/37)
2018	OLI	2018-10-12 (120/37), 2018-10-12 (120/38), 2018-10-12 (120/39),2018-10-12 (120/40),
		2018-10-03 (121/36), 2018-10-03 (121/37), 2018-10-03 (121/38), 2018-10-03 (121/39),
		2018-10-26 (122/36), 2018-10-26 (122/37), 2018-10-26 (122/38), 2018-10-26 (122/39),
		2018-10-17 (123/36), 2018-10-17 (123/37)

**Table 2:** Levels of prediction accuracy.

Level	Posterior Difference Ratio (C)	Small Error Possibility (P)
1	0.35	0.95
2	0.50	0.80
3	0.65	0.70
4 (Unqualified)	0.80	0.60

**Table 3:** Rules of land use type conversion under the ecological optimization scenario.

Type	Paddy Field	Unirrigated Field	Forest Land	Grass Land	Water Area	Wet Land	Built-Up Land
Paddy field	1	1	1	1	1	1	1
Unirrigated field	1	1	1	1	1	1	1
Forest land	0	0	1	0	1	1	0
Grass land	0	0	1	1	1	1	0
Water area	0	0	0	0	1	0	0
Wet land	0	0	0	0	1	1	0
Built-up land	1	1	1	1	1	1	1

**Table 4:** Driving factors of land use change.

Driving Factor	Name	Type of Data
Socio-economic factor	GDP	Continuous
	Population density	Continuous
Natural environment factor	DEM	Continuous
	Slope	Multi-class
	Average annual temperature	Continuous
	Average annual rainfall	Continuous
	Soil sand content	Multi-class
	Soil powder content	Multi-class
	Soil clay content	Multi-class
	Soil erosion	Multi-class
Traffic location factor	Distance to railway	Continuous
	Distance to expressway	Continuous
	Distance to highway	Continuous
	Distance to river	Continuous
	Distance to town	Continuous
	Distance to rural settlement	Continuous

**Table 5:** ROC values of the logistic regression model.

Type	Intercept	ROC Value
Paddy field	−5.5493	0.9187
Unirrigated field	−8.1118	0.9592
Forest land	−0.9745	0.9632
Grass land	−1.1348	0.8912
Water area	−19.1129	0.9586
Wet land	−23.0019	0.9299
Built-up land	−5.4330	0.8655

**Table 6:** ESV coefficients of different ecological service types for each land use type in Anhui Province (USD/ha/year).

Ecosystem Service	Type	Paddy Field	Unirrigated Field	Forest Land	Grass Land	Water Area	Wet Land	Built-Up Land	Unused Land
Provisioning services	Food production	356.78	222.99	76.08	99.69	209.87	133.79	2.62	0.00
	Raw material production	23.61	104.94	173.14	146.91	60.34	131.17	0.00	0.00
	Water supply	−689.96	5.25	89.20	81.33	2174.80	679.46	−1970.18	0.00
Regulating services	Gas regulation	291.20	175.77	569.28	516.81	202.00	498.45	−634.86	5.25
	Climate regulation	149.53	94.44	1705.21	1366.79	600.76	944.43	0.00	0.00
	Hydrological regulation	713.57	70.83	1243.49	1002.14	26,821.69	6356.51	0.00	7.87
	Environmental purification	44.60	26.23	506.32	451.23	1455.99	944.43	−645.36	26.23
Supporting services	Soil formation and retention	2.62	270.21	695.20	629.62	243.98	606.01	5.25	5.25
	Maintain nutrient cycling	49.84	31.48	52.47	47.22	18.36	47.22	0.00	0.00
	Biodiversity protection	55.09	34.10	632.24	571.90	668.97	2064.62	89.20	5.25
Cultural services	Recreation and culture	23.61	15.74	278.08	251.85	495.82	1240.87	2.62	2.62
	Total	1020.50	1051.99	6020.71	5165.48	32,952.59	13,646.95	−3150.71	52.47

**Table 7:** Prediction results comparison of land use structure for 2015 and 2018.

	2015			2018		
Type	Prediction Value (km^2^)	Actual Value (km^2^)	Difference (%)	Prediction Value (km^2^)	Actual Value (km^2^)	Difference (%)
Paddy field	41,666.11	41,483.58	0.44	41,133.32	41,108.65	0.06
Unirrigated field	35,485.84	35,446.85	0.11	35,270.74	35,095.26	0.50
Forest land	32,074.64	32,039.40	0.11	32,021.62	32,002.42	0.06
Grass land	8302.01	8283.79	0.22	8274.11	8285.71	−0.14
Water area	6429.65	6513.68	−1.29	6629.84	6683.98	−0.81
Wet land	1028.66	983.80	4.56	993.72	969.29	2.52
Built-up land	15,200.21	15,383.28	−1.19	15,984.16	15,992.16	−0.05

**Table 8:** Posterior difference ratio (C), small error possibility (P) and rediction accuracy level of land use demand.

Type	C	P	Accuracy Level
Paddy field	0.19	1.00	1
Unirrigated field	0.20	1.00	1
Forest land	0.39	0.92	2
Grass land	0.30	1.00	1
Water area	0.38	1.00	2
Wet land	0.56	0.75	3
Built-up land	0.16	1.00	1

**Table 9:** Prediction results comparison of land use structure for 2015 and 2018.

	2015			2018		
Type	Prediction Value (km^2^)	Actual Value (km^2^)	Difference (%)	Prediction Value (km^2^)	Actual Value (km^2^)	Difference (%)
Paddy field	42,649.47	41,483.58	2.81	41,068.75	41,108.65	−0.10
Unirrigated field	35,898.24	35,446.85	1.27	35,446.85	35,095.26	1.00
Forest land	31,886.70	32,039.40	−0.48	31,719.00	32,002.42	−0.89
Grass land	8342.97	8283.79	0.71	8200.95	8285.71	−1.02
Water area	6185.98	6513.68	−5.03	6513.68	6683.98	−2.55
Wet land	1089.83	983.80	10.78	993.63	969.29	2.51
Built-up land	13,410.43	15,383.28	−12.82	15,229.44	15,992.16	−4.77

**Table 10:** Comparison of the prediction accuracy between the GM (1,1) model and the Markov model.

	Type	Paddy Field	Unirrigated Field	Forest Land	Grass Land	Water Area	Wet Land	Built-Up Land
Difference (%)	2015	2.37	1.16	0.59	0.49	3.74	6.22	11.63
	2018	0.16	0.5	0.95	0.88	1.74	−0.01	4.72

**Table 11:** Prediction results of land use structure in the study area for 2025 and 2030 (Unit: km^2^).

Type	2025	2030
Paddy field	40,251.45	39,555.06
Unirrigated field	34,691.73	34,268.22
Forest land	31,963.12	31,903.61
Grass land	8260.34	8238.71
Water area	6782.26	6932.36
Wet land	954.93	927.83
Built-up land	17,997.14	19,667.92

**Table 12:** Accuracy assessment of the land use simulation results in 2018.

Actual Land Use Type									
Type	Paddy Field	Unirrigated Field	Forest Land	Grass Land	Water Area	Wet Land	Built-Up Land	Total	User’s Accuracy
Paddy field	9601	98	256	47	80	18	353	10453	0.9190
Unirrigated field	116	8511	39	25	32	11	254	8988	0.9472
Forest land	296	28	7658	94	17	3	31	8127	0.9424
Grass land	32	20	107	1779	5	0	12	1955	0.9100
Water area	78	51	15	7	1430	23	13	1617	0.8854
Wet land	12	2	2	1	35	194	2	248	0.7823
Built-up land	351	253	36	7	17	6	2910	3580	0.8128
Total	10,486	8963	8113	1960	1616	255	3575	34,968	
Producer’s accuracy	0.9156	0.9496	0.9438	0.9077	0.8849	0.7608	0.8140		
Total accuracy: 91.75%					Kappa coefficient: 0.8935				

**Table 13:** Simulation results of land use change in 2025 and 2030.

	2025		2030	
Type	Area (km^2^)	Proportion (%)	Area (km^2^)	Proportion (%)
Paddy field	40,251.44	28.77	39,555.08	28.28
Unirrigated field	34,691.72	24.80	34,268.24	24.50
Forest land	32,545.68	23.27	32,463.44	23.21
Grass land	8260.36	5.90	8238.72	5.89
Water area	6782.28	4.85	6932.36	4.96
Wet land	954.92	0.68	927.84	0.66
Built-up land	16,402.08	11.73	17,502.8	12.51

**Table 14:** ESV estimation results of Anhui Province (Unit: 10^7^ USD, %).

Type	Content	1995	2000	2005	2010	2015	2018	2025	2030
Paddy field	ESV	262.79	274.56	255.77	291.13	320.65	332.03	317.78	321.00
	Proportion	9.04	8.93	8.89	8.79	8.55	8.43	8.20	8.07
Unirrigated field	ESV	225.84	238.17	222.33	255.15	282.44	292.20	282.33	286.67
	Proportion	7.77	7.75	7.72	7.70	7.53	7.42	7.28	7.20
Forest land	ESV	1124.34	1193.68	1118.34	1297.11	1461.09	1524.95	1515.89	1554.27
	Proportion	38.68	38.84	38.85	39.15	38.98	38.73	39.11	39.06
Grass land	ESV	250.36	265.75	248.76	288.26	324.10	338.74	330.09	338.42
	Proportion	8.61	8.65	8.64	8.70	8.65	8.60	8.52	8.50
Water area	ESV	1158.27	1233.39	1163.45	1363.49	1625.77	1743.21	1728.98	1816.58
	Proportion	39.85	40.14	40.42	41.16	43.37	44.28	44.61	45.65
Wet land	ESV	86.52	92.24	86.14	100.49	101.69	104.69	100.82	100.69
	Proportion	2.98	3.00	2.99	3.03	2.71	2.66	2.60	2.53
Built-up land	ESV	−201.47	−224.72	−216.30	−282.62	−367.11	−398.79	−399.79	−438.53
	Proportion	−6.93	−7.31	−7.51	−8.53	−9.79	−10.13	−10.31	−11.02
Unused land	ESV	0.00	0.00	0.00	0.00	0.01	0.01	0.00	0.00
	Proportion	0.00	0.00	0.00	0.00	0.00	0.00	0.00	0.00
Total	ESV	2906.65	3073.06	2878.50	3313.02	3748.65	3937.04	3876.09	3979.11
